# Supplementary material for: Neuronal SNCA transcription during Lewy body formation
Source: Acta Neuropathol Commun. 2023 Nov 23;11:185. doi: 10.1186/s40478-023-01687-7 (PMC10666428; doi:10.1186/s40478-023-01687-7)
Supplement: Supplementary file 1 — Additional file 1: Fig. S1. Method of outlining the region of interest and capturing the positive signals in RNAscope. Raw data image of the SN section in a case of Lewy body disease (A). Lookup Table strength of the cell-specific marker and DAPI channels are increased enough to make the level of autofluorescence of neuromelanin and/or lipofuscin pigments visible (B). Then, the cell body borders are traced by their signals as total cell area (C). DAPI channel is selected and the nuclear edge is outlined as the nucleus area (D). Phosphorylated-α-syn immunostaining channel is represented in green and selected. The edge of LB is drawn as LB area (E). Subsequently, returning the LUT parameters to the default settings, positive signals corresponding to SNCA transcripts above the threshold within the region of interest are captured by the NIS-Elements software (F). Red displays SNCA transcripts, magenta shows RBFOX3 transcripts, and blue exhibits DAPI. Scale bar represents 10 μm. Fig. S2. Method of outlining the region of interest and capturing synuclein-immunoreactive neurites in HALO. The SN, region of interest, is demarcated by a yellow line (A). A magnified view of the boxed area in A is presented in (B), with the captured area highlighted in red (C). Fig. S3. Immunohistochemistry for SYN-1 and 5G4 α-synuclein (α-syn) antibodies. SYN-1 antibody cross-reacts with the physiological monomeric α-syn and shows a synaptic pattern in both cases of controls (A, C, E, G) and LBD (B, D, F, H) in addition to revealing Lewy body and related pathology in the diseased substantia nigra (SN, D) and putamen (F). In contrast, the 5G4 antibody does not label the physiological synaptic staining in the SN and putamen in cases of control (C, G) nor of LBD (D, H) and highlights only the disease associated α-syn immunoreactivity in LBD (D, H). Immunostaining for SYN-1 (A, B, E, F) and 5G4 (C, D, G, H) anti-α-syn antibodies in the SN (A–D) and putamen (E–H). The scale bars represent 50 μm for [file 40478_2023_1687_MOESM1_ESM.docx]

**Additional file 1**

**Supplementary methods**

**Immunohistochemistry**

For immunohistochemistry, midbrain, basal ganglia, and amygdala (n = 5 each in cases of LBD and controls) were analyzed. To assess the presence of physiological monomeric α-syn protein, we utilized immunohistochemistry with two α-syn specific antibodies: SYN-1 (1:2000, BD Biosciences, Franklin Lakes, NJ) [1], which is known to cross-react with physiological monomeric α-syn and disease-associated one [1], and 5G4, which specifically targets disease-associated α-syn protein and does not exhibit immunoreactivity against physiological monomeric α-syn protein [1-3]. Antigen retrieval was performed using Dako PT Link with low pH solution and 80% formic acid for 5 minutes for 5G4 anti-α-syn antibody. According to the manufacturer's protocol, immunostaining was performed using Dako Autostainer Link 48 and EnVision FLEX+ Visualization System. Subsequently, all sections were counterstained with hematoxylin.

**Morphometry of RNAscope**

The region of interest (ROI) for the total cell body, nuclear, and LB area was manually delineated using the cell-specific markers and DAPI signal positivity that allowed the identification of the contour of the neuronal cytoplasm (**Fig. S1A**). First, the Lookup Table (LUT) strength of the cell-specific marker and DAPI channels was increased sufficiently to enhance the visibility of autofluorescence from neuromelanin and/or lipofuscin pigments (**Fig. S1B**). The borders of the cell body were then traced based on these signals as the total cell body area (**Fig. S1C**). Subsequently, DAPI channel was selected, and the nuclear edge was outlined as the nuclear area (**Fig. S1D**). Then, phosphorylated-α-syn immunostaining channel is chosen and the edge of LB is drawn as LB area (**Fig. S1E**). Finally, the LUT parameters were restored to normal settings and the NIS-Elements software captured the area positive for *SNCA* transcripts above the threshold within the ROI (**Fig. S1F**).


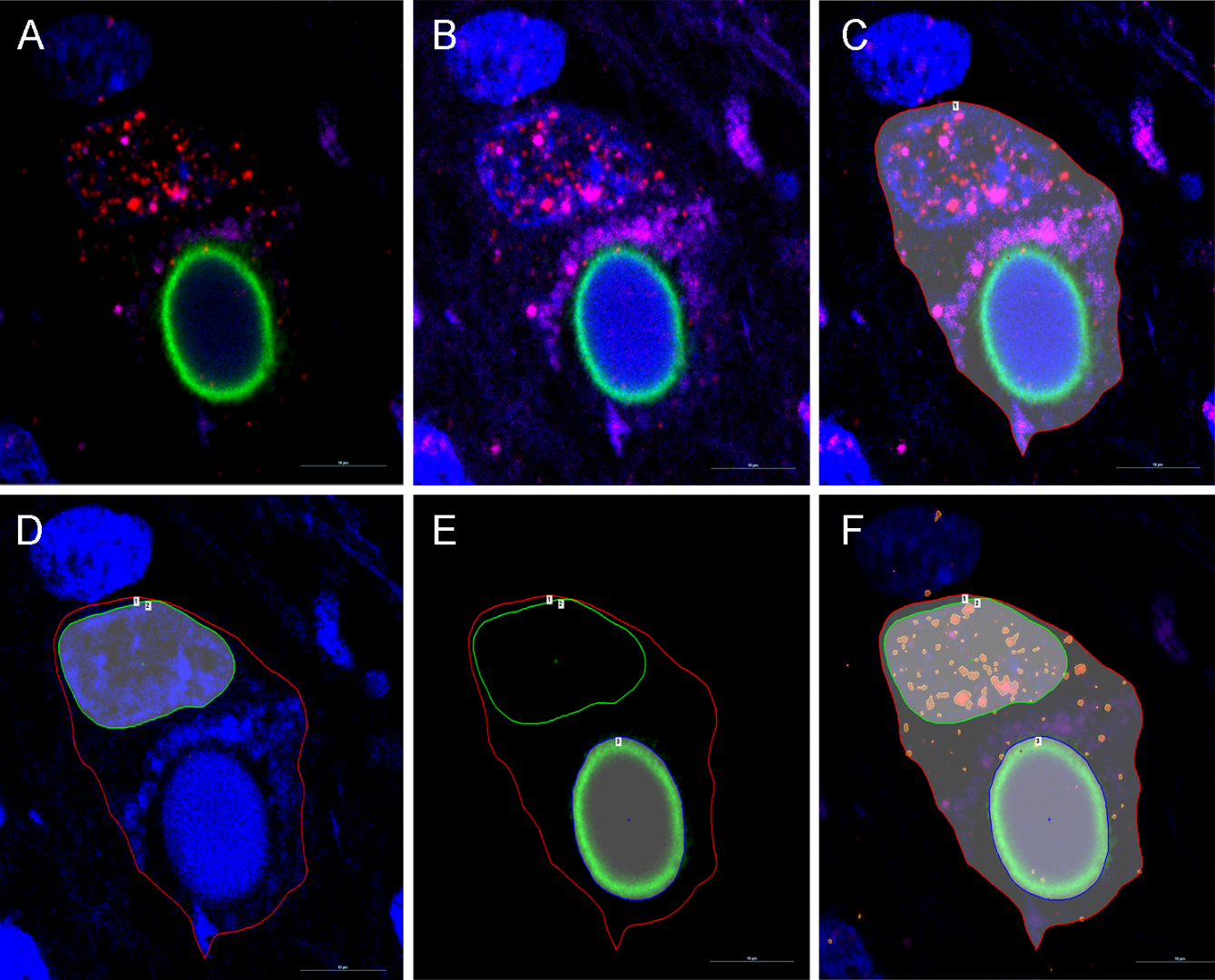


**Fig. S1. Method of outlining the region of interest and capturing the positive signals in RNAscope.**

Raw data image of the SN section in a case of Lewy body disease (**A**). Lookup Table strength of the cell-specific marker and DAPI channels are increased enough to make the level of autofluorescence of neuromelanin and/or lipofuscin pigments visible (**B**). Then, the cell body borders are traced by their signals as total cell body area (**C**). DAPI channel is selected and the nuclear edge is outlined as the nuclear area (**D**). Phosphorylated-α-syn immunostaining channel is represented in green and selected. The edge of LB is drawn as LB area (**E**). The NIS-Elements software captures the positive signals corresponding to *SNCA* transcripts above the defined threshold within the region of interest (**F**). Red displays *SNCA* transcripts, magenta shows *RBFOX3* transcripts, and blue exhibits DAPI. Scale bar represents 10 μm.

**Morphometry using HALO**

The SN was manually annotated, and an algorithm was established to exclusively quantify α-syn-immunoreactive neurites **(Fig. S2**). LBs and neuromelanin-containing neurons were not analyzed primarily due to the size of the objects and optical density considerations. Detailed parameters utilized in the HALO analysis can be found in **Table S1.**


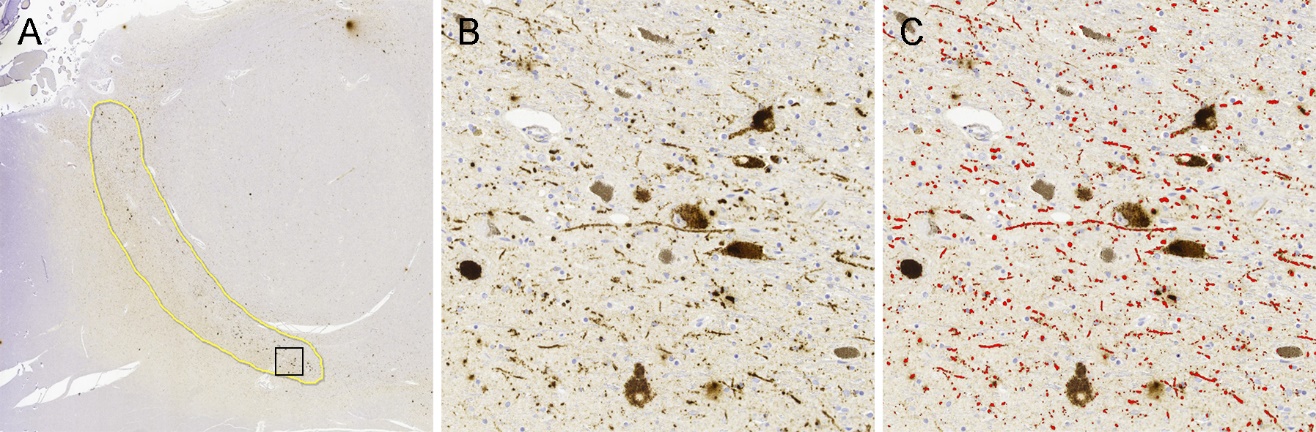


**Fig. S2 Method of outlining the region of interest and capturing synuclein-immunoreactive neurites in HALO**

The SN, region of interest, is demarcated by a yellow line (A). A magnified view of the boxed area in A is presented in (B), with the captured area highlighted in red (C).

**Table S1. HALO parameters**

| Parameters | Settings |
| --- | --- |
| Number of stains | 1 |
| Stain 1 | DAB |
| DAB stain color | 0.744, 0.944, 1.084 |
| Exclusion stain | None |
| DAB markup color | 255, 0, 0 |
| DAB blur radius | 1 |
| DAB contrast radius | 5 |
| DAB contrast threshold | 0.7 |
| DAB optical density | 0.5 |
| DAB object size | 0, 100 |
| DAB connect length | 0.5 |
| DAB fill holes | True |
| Output image | DAB markup |

**Supplementary results**

**Supplementary table 2. ANCOVA test regarding total cell body *SNCA* transcripts area density**

|  | **Estimated value** | **p** | **95% Confidence interval** |
| --- | --- | --- | --- |
| **Substantia nigra** |  |  |  |
| **α-syn morphologies** |  |  |  |
| Neurons without α-syn immunoreactivity | Reference | Reference | Reference |
| Punctate α-syn immunoreactivity | 0.507 | 0.892 | -6.817 to 7.83 |
| Compact irregular-shaped inclusion | -7.851 | 0.003 | -15.26 to -0.434 |
| Brainstem-type Lewy body | -13.878 | <0.001 | -21.338 to -6.418 |
| **Cases** |  |  |  |
| Case 1 | Reference | Reference | Reference |
| Case 2 | -10.219 | 0.001 | -16.403 to -4.035 |
| Case 3 | -9.19 | 0.029 | -17.414 to -0.966 |
| Case 4 | -8.872 | 0.019 | -16.29 to -1.454 |
| Case 5 | -4.986 | 0.129 | -11.433 to 1.46 |
| **Amygdala** |  |  |  |
| **α-syn morphologies** |  |  |  |
| Neurons without α-syn immunoreactivity | Reference | Reference | Reference |
| Punctate α-syn immunoreactivity | -2.711 | 0.104 | -5.983 to 0.561 |
| Brainstem-type Lewy body | -13.32 | <0.001 | -15.045 to -11.416 |
| **Cases** |  |  |  |
| Case 1 | Reference | Reference | Reference |
| Case 2 | 0.919 | 0.376 | -1.12 to 2.957 |
| Case 3 | -1.425 | 0.171 | -3.47 to 0.619 |

**Immunohistochemistry**

To support literature data on preserved α-syn protein expression in LBD, for demonstration we immunostained SN, striatum, and amygdala using an antibody that detects the physiological and disease-associated form of α-syn (SYN-1) and one that detects the disease-associated α-syn (5G4). In both control and LBD cases, SYN-1 immunoreactivity displayed a synaptic staining pattern in the SN, dorsal striatum, and amygdala as well as in LBs (**Fig. S3A, B, E, F**). On the other hand, disease-associated α-syn immunoreactivity using the 5G4 antibody was only detected in LBD cases (**Fig. S3C, D, G, H**).

**
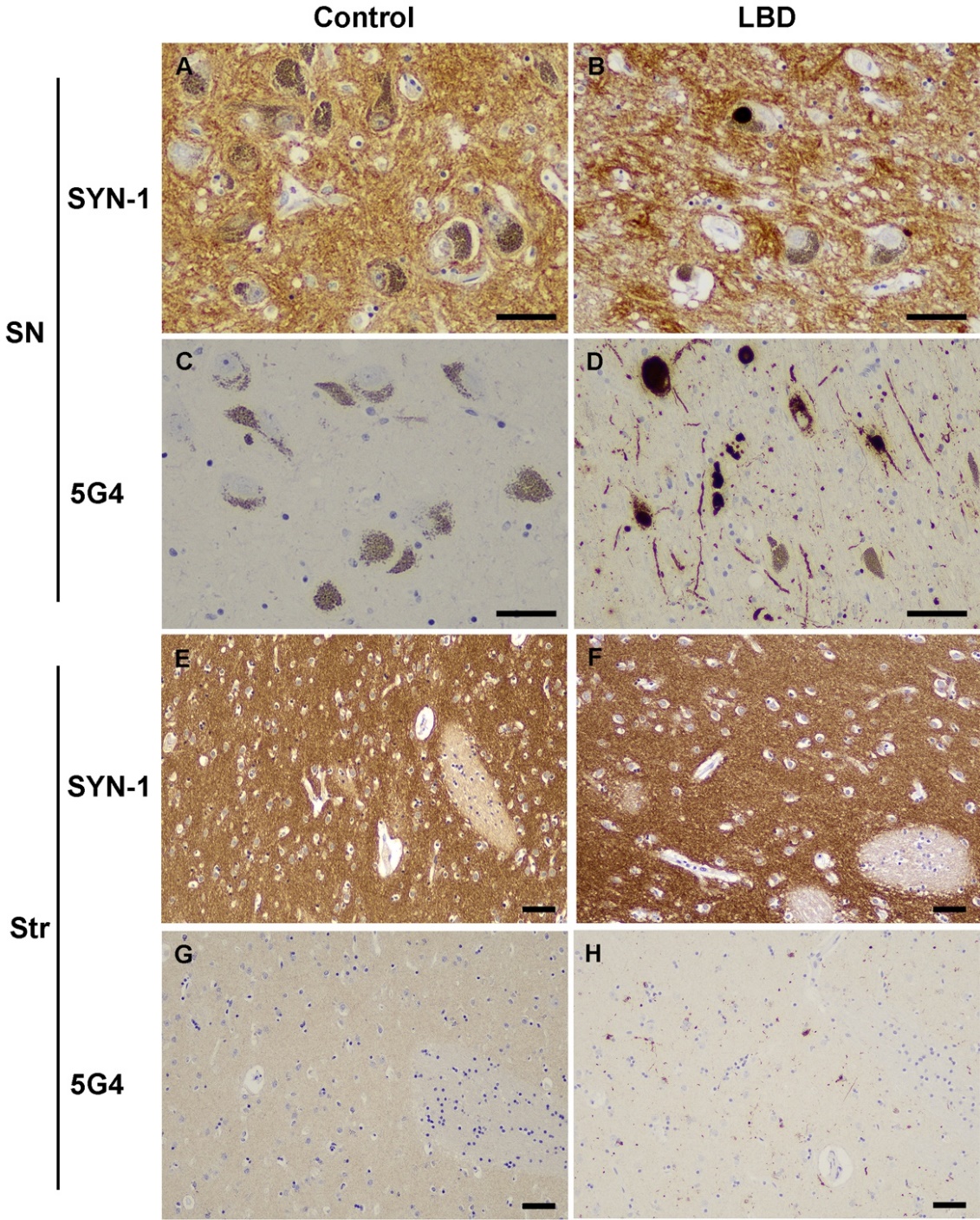
**

**Fig. S3. Immunohistochemistry for SYN-1 and 5G4 α-synuclein (α-syn) antibodies**

SYN-1 antibody cross-reacts with the physiological monomeric α-syn and shows a synaptic pattern in both cases of controls (**A, C, E, G**) and LBD (**B, D, F, H**) in addition to revealing Lewy body and related pathology in the diseased substantia nigra (**SN, D**) and putamen (**F**). In contrast, the 5G4 antibody does not label the physiological synaptic staining in the SN and putamen in cases of control (**C, G**) nor of LBD (**D, H**) and highlights only the disease associated α-syn immunoreactivity in LBD (**D, H**). Immunostaining for SYN-1 (**A, B, E, F**) and 5G4 (**C, D, G, H**) anti-α-syn antibodies in the SN (**A-D**) and putamen (**E-H**). The scale bars represent 50 μm for each image.

**The legends in Additional file 2. 3D RNAscope imaging of a brainstem-type Lewy body**

*SNCA* transcripts are rarely observed within phosphorylated-α-syn immunoreactive Lewy body areas. Green represents phosphorylated-α-syn immunostaining, red displays *SNCA* transcripts, magenta shows *RBFOX3* transcripts, and blue exhibits DAPI. See a video file, **Additional file 2**.

**Supplementary references**

1. Kumar ST, Jagannath S, Francois C, Vanderstichele H, Stoops E, Lashuel HA: How specific are the conformation-specific alpha-synuclein antibodies? Characterization and validation of 16 alpha-synuclein conformation-specific antibodies using well-characterized preparations of alpha-synuclein monomers, fibrils and oligomers with distinct structures and morphology. Neurobiol Dis. 2020;146:105086.

2. Kovacs GG, Wagner U, Dumont B, Pikkarainen M, Osman AA, Streichenberger N, et al: An antibody with high reactivity for disease-associated alpha-synuclein reveals extensive brain pathology. Acta Neuropathol. 2012;124(1):37-50.

3. Kovacs GG, Breydo L, Green R, Kis V, Puska G, Lorincz P, et al: Intracellular processing of disease-associated alpha-synuclein in the human brain suggests prion-like cell-to-cell spread. Neurobiol Dis. 2014;69:76-92.
